# Differential gene expression analysis using RNA-seq in the blood of goats exposed to transportation stress

**DOI:** 10.1038/s41598-023-29224-5

**Published:** 2023-02-03

**Authors:** Aditya Naldurtiker, Phaneendra Batchu, Brou Kouakou, Thomas H. Terrill, George W. McCommon, Govind Kannan

**Affiliations:** Agricultural Research Station, Fort Valley State University, 1005 State University Drive, Fort Valley, GA 31030 USA

**Keywords:** Biochemistry, Biotechnology, Cell biology, Molecular biology, Physiology

## Abstract

Transportation stress causes significant changes in physiological responses in goats; however, studies exploring the transcriptome of stress are very limited. The objective of this study was to determine the differential gene expressions and related pathways in the blood samples using RNA-seq procedure in Spanish goats subjected to different durations of transportation stress. Fifty-four male Spanish goats (8-mo old; BW = 29.7 ± 2.03 kg) were randomly subjected to one of three treatments (TRT; n = 18 goats/treatment): (1) transported for 180 min, (2) transported for 30 min, or (3) held in pens (control). Blood samples were collected before and after treatment for stress hormone, metabolite, and transcriptomic analysis. RNA-seq technology was used to obtain the transcriptome profiles of blood. Analysis of physiological data using SAS showed that plasma cortisol concentrations were higher *(P* < 0.01) in 180 min and 30 min groups compared to the control group. Enrichment analysis of DEGs related to transportation stress through Gene Ontology and KEGG databases revealed that the differentially expressed genes related to inflammatory pathways, caspases, and apoptosis such as *IL1R2, CASP14, CD14, TLR4*, and *MAPK14* were highly enriched in the transported group of goats compared to non-transported goats. Stress in goats leads to a sequence of events at cellular and molecular levels that causes inflammation and apoptosis.

## Introduction

Animals generally respond to stress with characteristic changes in physiology and behavior. During the alarm phase of the stress response, there is rapid release of certain catecholamines, increases in heart and respiratory rates, and elevation of body temperature^1^. Stress can also cause behavioral changes such as increased awareness, improved cognition, and enhanced analgesia^2^. During the restorative phase, the animal’s responses to stress are adaptive in nature and involve the secretion of corticotropin-releasing factor (CRF) from the hypothalamus. The CRF stimulates the pituitary gland to release adrenocorticotropic hormone, resulting in the activation of the adrenal cortex to synthesize glucocorticoids^2^

Glucocorticoids are good indicators of the reaction of animals to any kind of environmental change^3–5^. Results from several studies have shown that transportation stress increases adrenal cortisol activity, which modulates the immune system, resulting in an increase in total leukocyte count in the blood due to increases in the numbers of neutrophils and eosinophils^6,7^. Previous studies have shown that stress also increases plasma glucose and non-esterified fatty acid (NEFA) concentrations and creatine kinase activities in goats^3,8,9^. Stress causes a rapid increase in plasma glucose and NEFA concentrations mainly due to the release of catecholamines and the consequent increase in glycogenolysis and lipolysis^8,10^. Numerous other blood metabolites are also affected due to stress in goats. Batchu et al.^11^ reported that at the metabolome level, transportation stress significantly affected 21 amino acids and 18 acylcarnitines and other organic acids, with a majority of amino acids decreasing and acylcarnitines increasing over transportation time.

Among several transcriptome profiling methods, RNA-seq is the first sequencing-based method that allows the entire transcriptome to be surveyed in a very high throughput and quantitative manner. Transcriptomes of various stressors in different livestock species have been studied to some extent which has helped researchers gain valuable insights. For example, transcriptomic profiling of pigs subjected to stress has indicated that numerous genes related to stress response, including P53 signaling (tumor suppressor), TGF-β signaling (transforming growth factor), apoptosis signaling, and MAPK (mitogen-activated protein kinase) signaling pathways are significantly enriched^12^. In Holstein calves subjected to thermal stress, transcriptomic analysis has shown that TNF signaling pathway (tumor necrosis factor) and MAPK signaling pathway are significantly enriched^13^, and in Hu breed of sheep, MAPK signaling, PI3K-Akt (phosphatidylinositol 3-kinase and protein kinase B) signaling, and cAMP signaling pathways are enriched in response to thermal stress^14^. Fang et al.^15^ reported that pathways such as MAPK signaling, PI3K-Akt, and protein processing in the endoplasmic reticulum were enriched in Holstein dairy cattle when exposed to heat stress. Xu et al.^16^ observed that *FOS* and *MAPK1* were significantly upregulated among other differentially expressed genes (DEGs) in response to cold stress in Sanhe cattle. The authors suggested that *MAPK1* can be a candidate gene for cold stress in animals. In magpies, Wang et al.^17^ noted that the females are more sensitive to stress and energy imbalance as evidenced by upregulated of DEGs such as *JNKs* (c-Jun N-terminal kinases) and *AMPK* (5′AMP-activated protein kinase). These studies have provided valuable information on specific livestock species and experimental conditions that have added to the body of knowledge on transcriptomic profiles of stress in livestock.

Transcriptomic analysis of transportation stress in goats has not been conducted so far, despite the steady growth of the meat goat industry worldwide. As transportation is one of the most potent and unavoidable stressors in meat goat production, a thorough understanding of stress responses at the transcriptome level is essential. Identification of DEGs in response to this common stressor will increase our comprehension of the underlying mechanisms involved that are directly or indirectly reflected upon the physiology, behavior, health, and productivity of goats and allow development of suitable management methods and breeding strategies to minimize the deleterious effects. The overall aim of this study was to determine the differential gene expressions and related pathways in blood using RNA-seq procedure in Spanish goats subjected to either transportation for 180 min, transportation for 30 min, or no transportation but held in pens as controls. These two treatments were selected to simulate commercial practice as meat goats are typically transported for a period not exceeding 3 h prior to processing in the US.

## Methods

### Animals

This study was conducted in accordance with the ARRIVE guidelines (https://www.nc3rs.org.uk/arrive-guidelines). The protocols for this research were approved by the Animal Care and Use Committee at Fort Valley State University prior to beginning of the experiment. A total of fifty-four uncastrated male Spanish goats (8-mo old; BW = 29.7 ± 2.03 kg) were randomly subjected to one of three treatments (TRT; n = 18 goats/treatment): (1) transported for 30 min (approx. 50 km), (2) transported for 180 min (approx. 200 km), or (3) held in pens (control), on two different days. A livestock trailer with internal floor dimensions of 4.5 × 1.8 m were used to transport the goats. The ambient temperatures were − 3.0 ± 1.0 °C and 1.0 ± 1.0 °C on day 1 and 2, respectively. Prior to the experiment, goats were primarily raised on free range pasture with a grain supplement and with ad libitum access to hay and water. Feed was withheld overnight prior to the day of the experiment.

### Blood sampling

Blood samples were collected by trained personnel just before and after transportation prior to slaughter from the jugular vein using disposable needles and vacutainer tubes with anticoagulant EDTA. Blood samples collected were kept on ice, and later vacutainer tubes were centrifuged to separate plasma. Duplicate blood samples (5 mL) were also collected separately after transportation in PAXgene^®^ blood RNA tubes (PreAnalytiX, Qiagen, Germantown, MD) for RNA extraction.

### Cortisol

A commercially available kit (Cortisol ELISA Kit, Abnova, Taipei, Taiwan) was used to analyze plasma cortisol concentrations following the instructions provided by the manufacturer. Briefly, microplates (96-wells) were coated with 25 μL of goat plasma samples followed by the addition of 100 μL of cortisol enzyme conjugate solution to each sample. After 1 h of incubation at 37 °C, the plates were washed four times, 100 μL of color reagent (3,3′, 5,5′ -tetramethylbenzidine, TMB) added to each well, and then 50 μL of the stop solution was added to stop the reaction. Absorbances were measured at 450 nm using a Synergy HTX Microplate Reader (Bio-Tek, Winooski, VT). The cortisol concentrations were determined against a standard curve. This sheep cortisol kit has been validated for goats in our laboratory. The minimum detectable concentration of cortisol by this method is 1.0 ng/mL.

### Metabolites

The Stanbio Glucose Liqui-UV (Hexokinase) Kit (Stanbio Laboratory, Boerne, TX) was used to determine plasma glucose concentrations following the manufacturer’s instructions. Plasma non-esterified fatty acid (NEFA) concentrations were also determined using a commercial kit (NEFA-HR (2) Kit, Fujifilm, Mountain View, CA) according to the manufacturer’s instructions. Plasma creatine kinase concentrations were determined using a Creatine Kinase Assay Kit (Abnova Corporation, Tapei, Taiwan). The kit is based on enzyme coupled reactions in which creatine phosphate and adenosine diphosphate (ADP) are converted to creatine and adenosine triphosphate (ATP) by the CK enzyme. The generated ATP is used to phosphorylate glucose by hexokinase to generate glucose-6-phosphate, which is then oxidized by NADP in the presence of glucose-6-phosphate dehydrogenase (G6P-DH). The NADH produced is proportional to the CK activity in the given plasma sample.

### Differential leukocyte counts

Blood samples were collected separately in 3 mL vacutainer tubes coated with EDTA (K3) for differential leukocyte counts. Neutrophil (N), lymphocytes (L), monocytes and eosinophil counts were determined using a VetScan HM5 Haemotology Analyzer (Abaxis, Union city, CA) according to manufacturer’s instructions.

### Blood RNA extraction

The PAXgene^®^ blood RNA tubes were transferred to a − 80 °C freezer for RNA extraction. Blood RNA was extracted using a MagMAX™ Stabilized Blood Tubes RNA Isolation Kit by applied biosystems (Thermo Fisher Scientific, Waltham, MA) according to manufacturer’s instructions.

### RNA-Seq library preparation and sequencing

A NanoDrop spectrophotometer (IMPLEN, CA, USA) was used to check the purity of RNA and any degradation and contamination were monitored on 1% agarose gels. The RNA Nano 6000 Assay Kit of the Bioanalyzer 2100 system (Agilent Technologies, CA, USA) was used for RNA integrity and quantification assessment. For mRNA isolation, RNA-seq library preparation, and sequencing procedures, 27 samples were shipped to Novogene Corporation Inc. (Sacramento, CA, USA). The methodologies described in this section and under quality control, reads mapping and gene expression analysis, and statistical analysis sections are abbreviated versions of those provided by Novogene Corporation Inc. For RNA sample preparation, a total of 1 µg RNA per sample was used as input material. After generating the sequencing libraries using the NEBNext^®^ Ultra**™** RNA Library Prep kit (Illumina^®^, NEB, USA), index codes were added to attribute sequence to each sample. Using poly-T oligo-attached magnetic beads, the mRNA was purified from total RNA. For fragmentation of mRNA, divalent cations under elevated temperature in NEBNext First Strand Synthesis Reaction Buffer were used. Random hexamer primer and M-MuLV Reverse Transcriptase RNaseH were used to synthesize the first strand of cDNA. Then DNA polymerase I and RNase H were used to synthesize the second strand of cDNA. Adapters were ligated to generate 250–300 bp length fragments after adenylation of 3’ ends of cDNA fragments. Then, PCR was conducted using Phusion High-Fidelity DNA polymerase, Universal PCR primers and Index (X) primer. An AMPure XP system (Beckman Coulter, Beverely, MA, USA) was used for cDNA purification and an Agilent Bioanalyzer 2100 system was used for cDNA library quality assessment. To cluster the index-coded samples on a cBot Cluster Generation System, a PE Cluster Kit cBot-HS (Illumina) was used. Then, library preparations were sequenced, and paired-end reads were generated. In general, only for whole blood samples, we will remove the globin mRNA in the process of lib prep. Because blood samples contain abundant globin mRNA, this portion was removed to reduce the background noise during library preparation, which is a routine procedure followed for blood samples at Novogene labs.

### Quality control

Raw data (raw reads) of FASTQ^18^ format were initially processed through fastp. In this step, clean data (clean reads) were obtained by removing reads containing adapter and poly-N sequences (N > 10%) and reads with low quality from raw data. Simultaneously, Q20 (the proportion of bases with a phred base quality score of Q20; the proportion of read bases whose error rate was less than 1%), Q30 (the proportion of bases with a phred base quality score of Q30; the proportion of read bases whose error rate was less than 0.1%) and GC content (the proportion of G and C base numbers of the total bases) of the clean data were calculated. All downstream analyses were based on clean data.

### Reads mapping on the caprine reference genome and gene expression analysis

Reads were mapped either to genome or transcriptome with a reference sequence. For the significant expression of differentially expressed genes, reads were mapped to transcriptome directly. However, alignment was carried out at genomic level in the case of alternative splicing. While mapping to transcriptome or genome, reads could be uniquely assigned to a particular position or could be multi-mapped, and this required a gapped mapper that can span across splice junctions^19^. All reference genome and gene annotation files were downloaded directly from the genome website browser (NCBI/UCSC/Ensembl). Clean paired-end reads were mapped directly to the reference genome of goat (*Capra hircus*) using the software HISAT2, which utilizes a large set of small GraphFM (GFM) indexes that completely mask the entire genome. Novel genes and transcripts were identified by the software Stringtie which assembled the set of transcript isoforms of each BAM file obtained in the mapping step. Gffcompare was used to compare the Stringtie assemblies to the annotation files that helped to sort out the new genes from the known ones. Transcript abundance reflected the gene expression levels directly. Gene expression levels were estimated by the transcript abundance that mapped to the genome or exon. Read counts were proportional to gene expression levels, gene length, and sequencing depth. Transcript abundances were estimated as reads per kilobase of exon model per million mapped reads (RPKM), which was calculated based on the length of the gene and reads count mapped to the genes.

Prior to differential gene expression analysis, for each sequenced library, the read counts were normalized^20^ with the scaling factors from the Trimmed means of M-values (TMM) using edgeR R package. Differentially expressed genes of two conditions were detected using the edgeR R package^21^. Differential gene expression analysis for two conditions (three biological replicates per condition) was carried out using DESeq2^22^. Hierarchical clustering was used to sort out the genes with similar expression patterns under different experimental conditions. In addition to the fragments per kilobase of transcript per million fragments mapped (FPKM) cluster^23^, the H-Cluster was used to cluster the log2 (fpkm + 1) for gene expression pattern. The resulting *P*-values were adjusted using Benjamini and Hochberg’s approach for controlling false discovery rate (FDR)^24^. The fold changes (in log_2_ scale), *P* values and q-values (corrected *p*-values) of DEGs were reported from the files provided from DESeq2. Genes with a *q*-value of < 0.05 and fold change > 1.0 were considered to be significantly expressed.

Gene ontology enrichment analysis was executed by the ClusterProfiler R package, which corrected the gene length bias. Statistical enrichment of differentially expressed genes in Kyoto Encyclopedia of Genes and Genomes (KEGG) pathway was analyzed by ClusterProfiler R package^25^. To avoid overly narrow categories, functional categories with more than 20 genes were tested. In addition, the procedures described by Benjimini and Hochberg^24^ were used for multiple testing. Functional categories with FDR ≤ 0.05 were considered significant. Enrichment of GO terms and KEGG pathways with genes differentially expressed between the treatments were analyzed based on cumulative hypergeometric distribution^26^.

### Statistical analysis

Blood hormone and metabolite data were analyzed using General Models Procedure in SAS (release 9.1, SAS Institute, Cary, NC, USA) with TRT and day as independent variables. Pretreatment concentrations were used as covariates in the analysis. The data were first checked for normality and homogeneity of variances using Levene’s Test and Shapiro–Wilk’s Test. Whenever required the data were transformed to log scale to meet the assumptions of ANOVA. The data were then back-transformed and presented for ease of interpretation. When significant by ANOVA at *P* < 0.05, the means were separated using the LSD test.

### Ethics approval and consent to participate

The methods in this investigation were conducted according to relevant guidelines and regulations. The animal care protocols were approved by the FVSU Agricultural and Laboratory Animal Care and Use Committee prior to beginning of the study following the ADSA-ASAS-PSA Guide for Care and Use of Agricultural Animals in Research and Teaching.

## Results

### Blood hormone and metabolite concentrations

**Figure 1:**
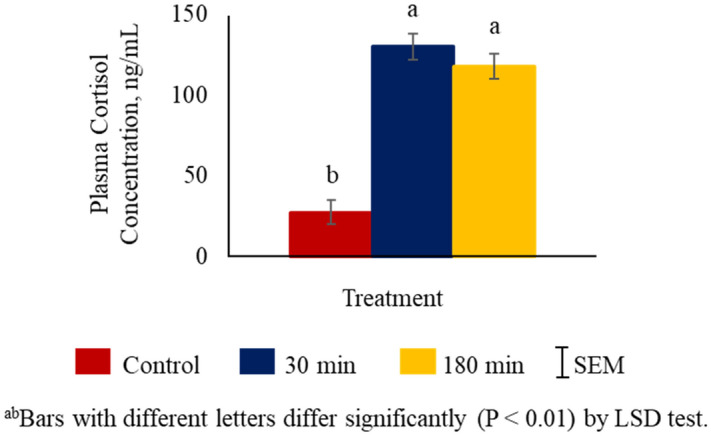
Effects of stress treatment (Control = not transported but held in pens; 30 min = transported for 30 min; 180 min = transported for 180 min) on mean ± SEM plasma cortisol concentrations in goats.

**Figure 2:**
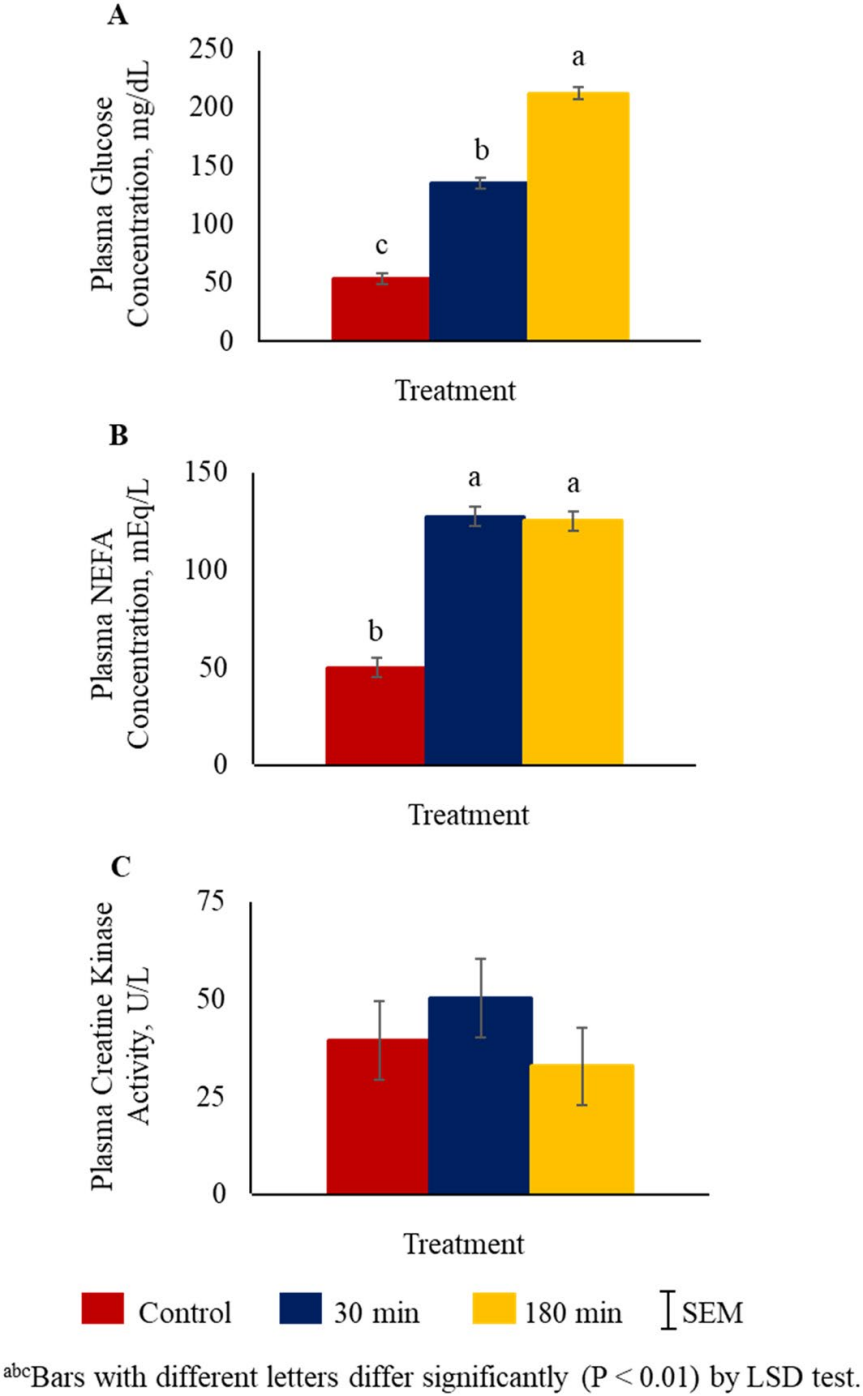
Effects of stress treatment (Control = not transported but held in pens; 30 min = transported for 30 min; 180 min = transported for 180 min) on mean ± SEM plasma (**A**) glucose and (**B**) non-esterified fatty acids (NEFA) concentrations, and (**C**) creatine kinase activity in goats.

Plasma cortisol concentrations were higher (*P* < 0.01) in the 30 min and 180 min transportation groups compared to the control group (Fig. 1). Glucose concentrations were the lowest in the control group, highest in the 180 min group, and intermediate in the 30 min group (*P* < 0.01; Fig. 2A). Plasma NEFA levels were higher (*P* < 0.01) in 30 min and 180 min groups compared to the control group (Fig. 2B), while plasma creatine kinase concentrations were not significantly influenced by TRT (Fig. 2C).

### Differential leukocyte counts

**Figure 3:**
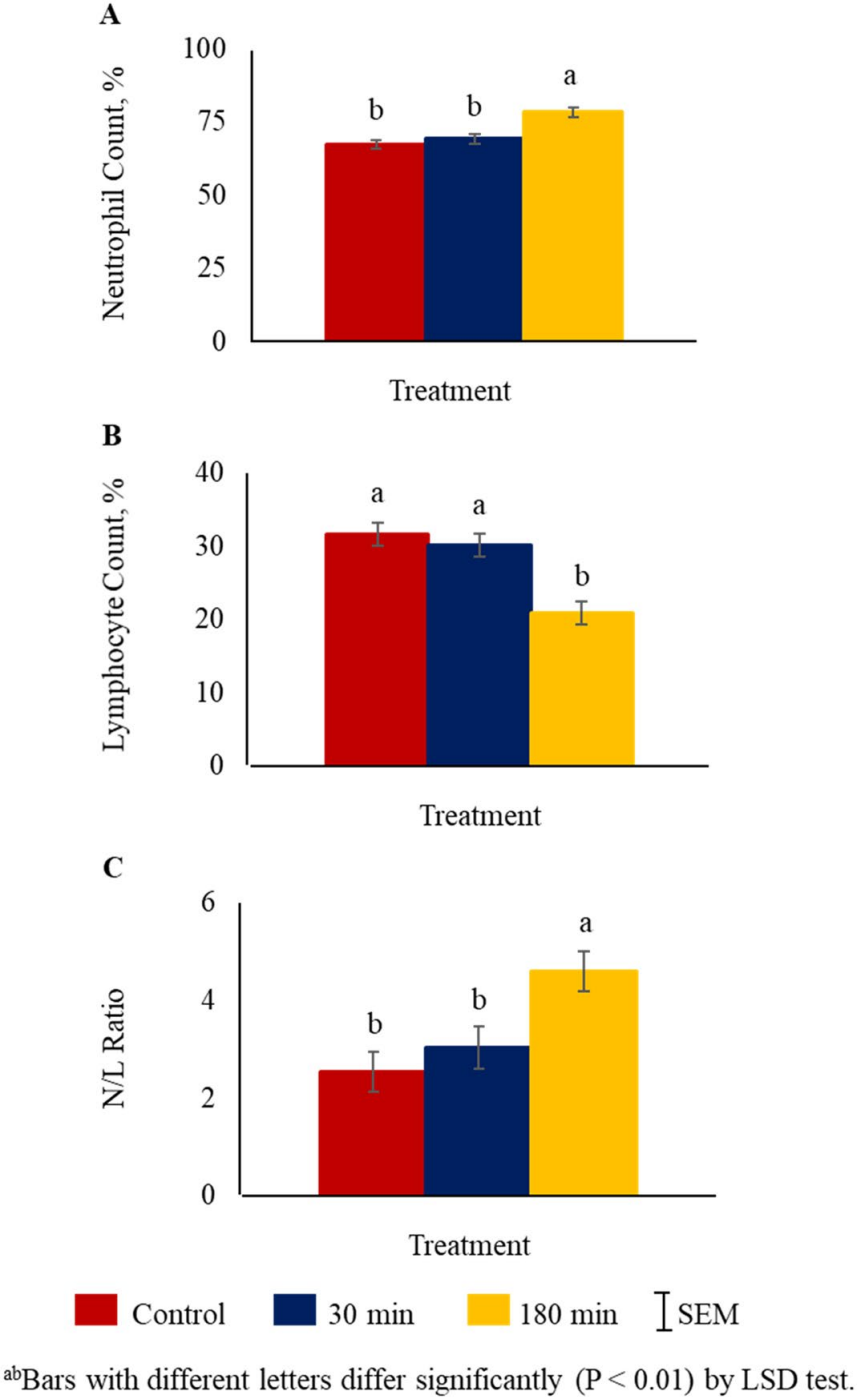
Effects of stress treatment (Control = not transported but held in pens; 30 min = transported for 30 min; 180 min = transported for 180 min) on mean ± SEM (**A**) neutrophil (N) and (**B**) lymphocyte (L) counts, and (C) N/L ratio in goats.

Treatment (*P* < 0.01) had a significant effect on neutrophil and lymphocyte counts and the N/L ratio. Neutrophil counts were higher (*P* < 0.01) in 180 min group compared to the 30 min or control groups (Fig. 3A). Lymphocyte counts were lower (*P* < 0.01) in 180 min group compared to the other two groups (Fig. 3B). The N/L ratio was higher (*P* < 0.01) in 180 min compared to control or 30 min TRT groups (Fig. 3C).

### Sequencing

A total of 72 cDNA libraries were constructed using blood samples. The summary of sequencing results is given in Table 1. From eighteen blood samples, an average of 23,634,716, 19,259,815, and 29,655,586 raw reads were obtained from control, 30 min, and 180 min groups, respectively. After filtering the raw reads with adapter sequences and low-quality reads, a total average of 22,253,472**,** 18,309,599, and 28,261,890 clean reads were obtained with phred base quality scores of Q20 > 97% and Q30 > 93%, and guanine-cytosine (GC) content > 54%.

**Table 1:** Summary of sequencing results of blood samples from goats subjected to transportation stress treatment (TRT).

TRT	Raw reads	Clean reads	Unique reads	Total mapped reads	Total mapping %	Q20^d^ %	Q30^e^ %	GC content^f^ %
Control^a^	23,634,716	22,253,472	35,758,497	40,481,450	90.73	97.07	93.11	55.01
30 min^b^	19,259,815	18,309,599	31,148,020	33,625,090	91.41	97.45	93.96	54.41
180 min^c^	29,655,586	28,261,890	46,892,790	52,644,352	93.06	97.70	94.08	54.87

^a^Not transported but held in pens;

^b^Transported for 30 min;

^c^Transported for 180 min;

^d^Phred values greater than 20 base number contain the percentage of total bases;

^e^Phred values greater than 30 base number contain the percentage of total bases;

^f^Percentage of guanine and cytosine base numbers of total bases.

### Gene expression

**Figure 4:**
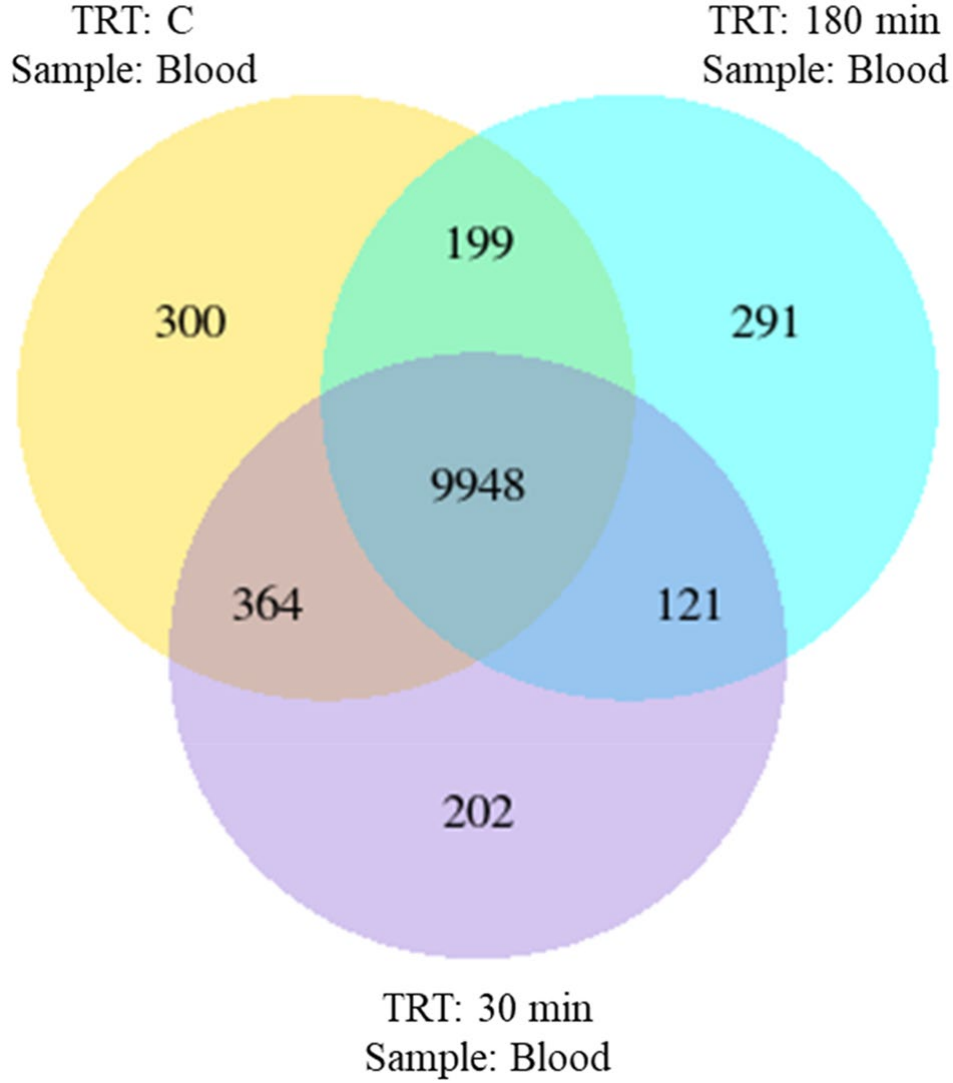
Venn diagram showing the co-expression of genes in blood samples from goats subjected to stress treatments (TRT: control, C = not transported but held in pens; 30 min = transported for 30 min; or 180 min = transported for 180 min).

The summaries of FPKM are given in Table 2. The summary of genes expressed in each treatment group and genes co-expressed are shown in Fig. 4. A total of 9948 genes were expressed commonly, with 199, 121, and 364 genes shared between control and 180 min, 180 min and 30 min, and control and 30 min groups, respectively.

**Table 2:** Fragments per kilobase of exon per million mapped reads (FPKM) of blood samples subjected to transportation stress.

Stress treatment	FPKM interval				
	0–1	1–3	3–15	15–60	> 60
Control^a^	17,110 (62.74%)	2573 (9.44%)	4432 (16.25%)	2262 (8.29%)	895 (3.28%)
30 min^b^	17,551 (64.36%)	2573 (9.44%)	4647 (17.05%)	2294 (8.41%)	874 (3.21%)
180 min^c^	16,884 (61.91%)	2474 (9.07%)	4033 (14.79%)	2165 (7.94%)	1048 (3.84%)

^a^Not transported but held in pens;

^b^Transported for 30 min;

^c^Transported for 180 min.

### Differential gene expression

**Figure 5:**
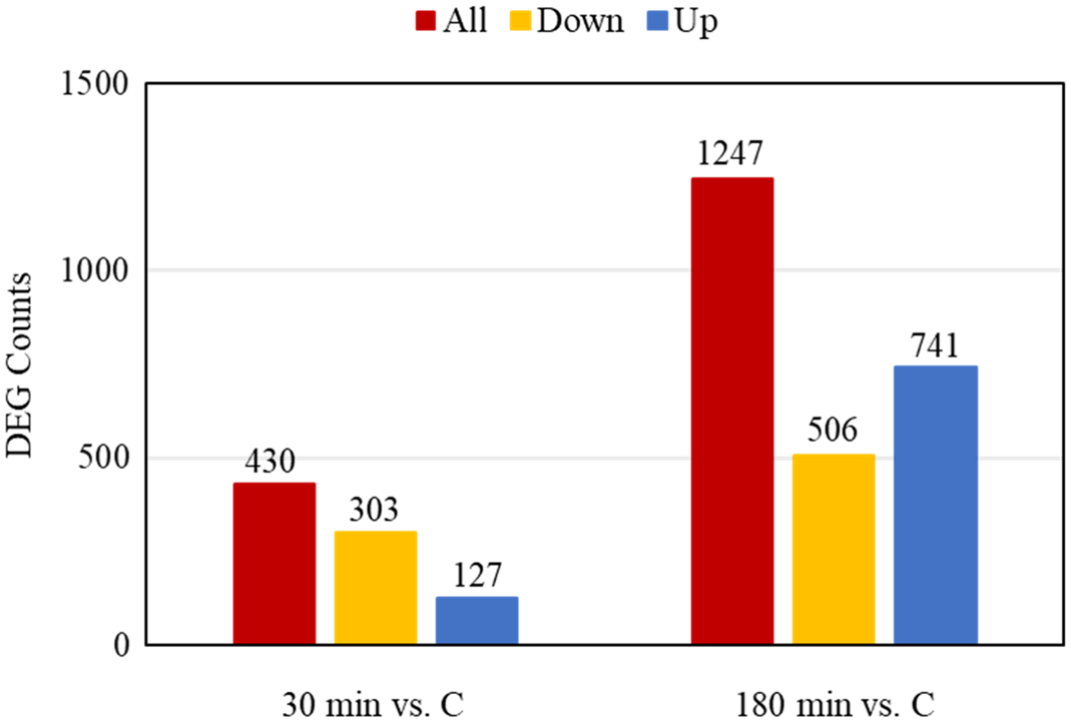
Differentially expressed genes of blood samples from goats subjected to stress treatments (TRT: control, C = not transported but held in pens; 30 min = transported for 30 min; or 180 min = transported for 180 min).

**Figure 6:**
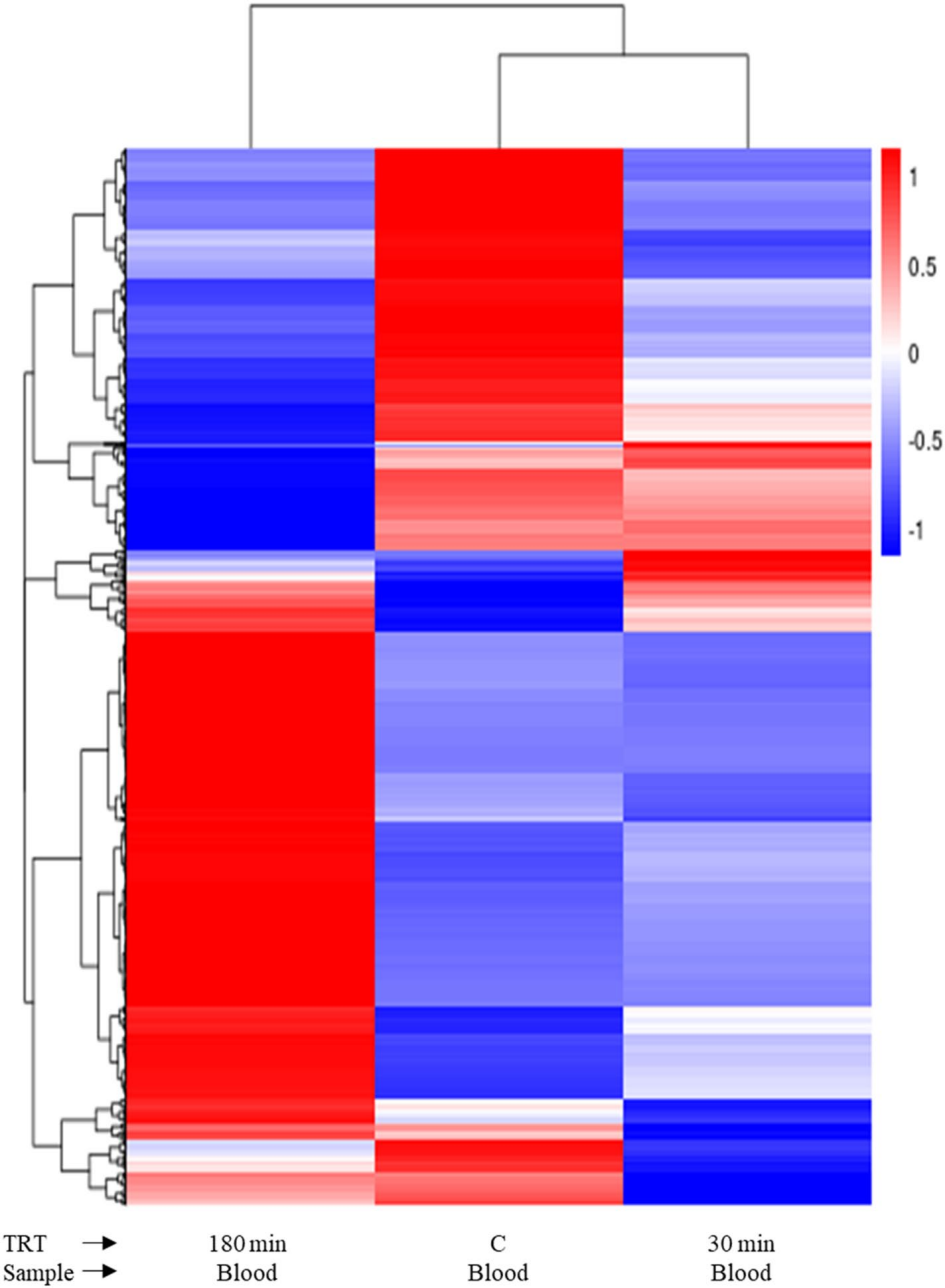
Heat map of differentially expressed genes in blood samples from goats subjected to stress treatments (TRT: control, C = not transported but held in pens; 30 min = transported for 30 min; or 180 min = transported for 180 min).

The counts of upregulated and downregulated DEGs in each comparison are shown in Fig. 5 and the patterns of DEGs in each treatment group were visualized using a heatmap (Fig. 6; Supplementary Fig. S1). The DESeq2 software identified a total of 430 DEGs in blood samples for control versus 30 min comparison, of which 303 genes were downregulated and 127 genes were upregulated (Fig. 5). In this comparison, DEGs such as *NFKBIA* (nuclear factor kappa B inhibitor alpha), *GADD45A* (growth arrest and DNA damage-inducible alpha), *CEBPB* (CCAAT/enhancer binding protein beta), *CXCR4* (C-X-C motif chemokine receptor 4), and *PTX3* (pentraxin 3) were significantly upregulated (*P* < 0.01) and *PRAG1* (PEAK1 related kinase-activating pseudokinase 1), *RARB* (retinoic acid receptor beta), *IQCG* (IQ motif containing G), *ZDHHC15* (zinc finger DHHC-type containing 15), and *KIF12* (kinesin family member 12) were significantly (*P* < 0.01) downregulated (Table 3). For 180 min vs control comparison of blood samples, 741 DEGs were upregulated and 506 DEG were downregulated (Fig. 5). In this comparison, DEGs such as *TLR4* (toll-like receptor 4), *TLR7* (*Capra hircus* toll-like receptor 7), *MAPK13* (mitogen-activated protein kinase 13), *CAMKK1* (calcium/calmodulin-dependent protein kinase kinase 1), and *PTX3* (pentraxin 3) were significantly (*P* < 0.01) upregulated and DEGs such as *IQCG* (IQ motif containing G), *PTGR1* (prostaglandin reductase 1), *RF00602* (small cajal body-specific RNA 21), *TNFRSF25* (TNF receptor superfamily member 25), and *LCK* (LCK proto-oncogene Src family tyrosine kinase) were significantly (*P* < 0.01) downregulated (Table 4). In addition, *CASP14* (caspase 14) among several caspases was significantly upregulated. The upregulated DEGs in both comparisons are related to apoptosis and inflammatory responses.

**Table 3:** Differentially expressed genes (DEG) in blood samples of goats transported for 30 min vs. goats not transported (control).

Gene ID	Log2fold change	*P* value	Gene name	Gene description
Upregulated				
ENSCHIG00000010157	1.695606137	5.62E−10	NFKBIA	NFKB inhibitor alpha
ENSCHIG00000025537	1.417557644	5.12E−08	GADD45A	Growth arrest and DNA damage inducible alpha
ENSCHIG00000026165	1.453109349	4.69E−07	CEBPB	CCAAT/enhancer binding protein beta
ENSCHIG00000013798	1.146338102	2.89E−06	CXCR4	C-X-C motif chemokine receptor 4
ENSCHIG00000012523	3.080881993	0.000023374	PTX3	Pentraxin 3
ENSCHIG00000011855	1.588728094	0.000090640	DDIT4	DNA damage inducible transcript 4
ENSCHIG00000018498	0.744120807	0.003231251	TLR4	Toll like receptor 4
ENSCHIG00000010803	4.495720604	0.009835413	CCL24	C–C motif chemokine ligand 24
ENSCHIG00000020142	2.721596928	0.011144163	CXCL8	C–X–C motif chemokine ligand 8
ENSCHIG00000004093	0.63351115	0.041689969	JUN	Jun proto-oncogene, AP-1 transcription factor subunit
Downregulated				
ENSCHIG00000011793	− 0.635822347	0.000182298	PRAG1	PEAK1 related, kinase-activating pseudokinase 1
ENSCHIG00000011887	− 6.614826723	0.000909012	RARB	Retinoic acid receptor beta
ENSCHIG00000010981	− 2.81799128	0.001304226	IQCG	IQ motif containing G
ENSCHIG00000012700	− 3.625486427	0.001472814	ZDHHC15	Zinc finger DHHC-type containing 15
ENSCHIG00000018125	− 1.665650728	0.001771593	KIF12	Kinesin family member 12
ENSCHIG00000027224	− 4.135466666	0.002768673	NXPH3	Neurexophilin 3
ENSCHIG00000020630	− 1.611893441	0.002836901	WNK4	WNK lysine deficient protein kinase 4
ENSCHIG00000009760	− 0.722057447	0.002993397	MBD4	Methyl-CpG binding domain 4, DNA glycosylase
ENSCHIG00000016682	− 1.211284104	0.003043671	DHRSX	Dehydrogenase/reductase X-linked
ENSCHIG00000006098	− 1.040520403	0.003193152	CERS4	Ceramide synthase 4

**Table 4:** Differentially expressed genes (DEG) in blood samples of goats transported for 180 min vs. goats not transported (control).

Gene ID	Log2fold change	*P* value	Gene name	Gene description
Upregulated				
ENSCHIG00000012523	5.776576685	1.01E−12	PTX3	Pentraxin 3
ENSCHIG00000019426	1.606853293	0.000025757	CAMKK1	Calcium/calmodulin dependent protein kinase 1
ENSCHIG00000023563	1.263688040	0.006030954	MAPK13	Mitogen-activated protein kinase 13
ENSCHIG00000018498	1.291476357	0.000697660	TLR4	Toll like receptor 4
ENSCHIG00000021012	0.791638027	0.009356838	TLR7	Capra hircus toll-like receptor 7 (TLR7), mRNA
ENSCHIG00000012735	0.791638027	0.003685898	TNFRSF1A	TNF receptor superfamily member 1A
ENSCHIG00000010803	3.601218185	0.004798859	CCL24	C–C motif chemokine ligand 24
ENSCHIG00000012371	1.272469567	0.015320533	BCL2L15	BCL2 like 15
ENSCHIG00000018930	1.905512852	0.015444315	CASP14	Caspase 14
ENSCHIG00000016438	0.844031117	0.016935532	BAX	BCL2 associated X, apoptosis regulator
Downregulated				
ENSCHIG00000010981	− 3.295723997	0.00003416	IQCG	IQ motif containing G
ENSCHIG00000026536	− 1.263181204	0.00004094	PTGR1	Prostaglandin reductase 1
ENSCHIG00000003555	− 5.173737307	0.00005095	RF00602	Small Cajal body-specific RNA 21
ENSCHIG00000010592	− 0.986663388	0.00005367	TNFRSF25	TNF receptor superfamily member 25
ENSCHIG00000013860	− 0.741645397	0.00005473	LCK	LCK proto-oncogene, Src family tyrosine kinase
ENSCHIG00000024398	− 2.182544992	0.000109134	INHBB	Inhibin beta B subunit
ENSCHIG00000026098	− 4.840444601	0.000193664	GPR179	G protein-coupled receptor 179
ENSCHIG00000011037	− 2.241685534	0.000198001	UCP1	Uncoupling protein 1
ENSCHIG00000026443	− 1.269291185	0.000407561	RAB37	RAB37, member RAS oncogene family
ENSCHIG00000026725	− 1.225373497	0.00043629	TUBB3	Tubulin beta 3 class III

### Gene ontology

Gene ontology functional annotations were carried out to identify the biological functions of DEGs. A total of 1677 DEGs were identified for two different treatment comparisons of blood samples. Comparison of 30 min transportation versus control group revealed that biological process (BP) terms related to inflammatory response, leukocyte migration, negative regulation of phosphorylation, leukocyte activation, and cytokine production were significantly (*P* < 0.01) upregulated and BP terms related to B cell receptor signaling pathway, regulation of G-protein coupled receptor protein signaling pathway, immune response-activating cell surface receptor signaling pathway, immune response-activating signal transduction, and cellular response to DNA damage stimulus were significantly (*P* < 0.01) downregulated (Table 5). For 180 min versus control comparison, significantly (*P* < 0.01) upregulated BP terms were inflammatory response, interleukin-6 production, regulation of defense process, cytokine production, and tumor necrosis factor production, and significantly (*P* < 0.01) downregulated BP terms were notochord development, collagen fibril organization, negative regulation of cell motility, regulation of nucleotide metabolic process, and response to lipopolysaccharide (Table 6).

**Table 5:** Upregulated and downregulated biological processes in blood samples of goats transported for 30 min vs. goats not transported (control).

Gene ontology ID	Description	*P* value	Gene name
Upregulated			
GO:0,006,954	Inflammatory response	6.7745E−06	NFKBIA, VNN1, HYAL2, PTAFR, TLR4, BST1, CCL24, SOCS3
GO:0,050,900	Leukocyte migration	0.00014867	ICAM1, C5AR1, BST1, CCL24, CXCL8, C5AR2, B4GALT1, SELL
GO:0,042,326	Negative regulation of phosphorylation	0.00045675	GADD45A, DDIT4, DUSP1, HYAL2, TLR4, SAMSN1, SOCS3, JUN
GO:0,045,321	Leukocyte activation	0.0012427	CEBPB, ICAM1, VNN1, HYAL2, TLR4, SAMSN1, CXCL8, PRDM1
GO:0,001,816	Cytokine production	0.00387164	CEBPB, HYAL2, PTAFR, TLR4, KLF4, C5AR1, C5AR2, LITAF
GO:0,010,942	Positive regulation of cell death	0.00725827	GADD45A, FasL, HYAL2, TLR4, B4GALT1, EGLN3, BTG1
GO:0,097,190	Apoptotic signaling pathway	0.01695727	CEBPB, DDIT4, FasL, ICAM1, VNN1, HYAL2, SRGN
GO:0,006,979	Response to oxidative stress	0.00150704	FOS, VNN1, HYAL2, TLR4, TXNIP, MGST1, JUN
GO:0,051,347	Positive regulation of transferase activity	0.12942290	GADD45A, TLR4, KLF4, RALB, ARRDC4
GO:0,045,859	Regulation of protein kinase activity	0.22611585	GADD45A, DUSP1, HYAL2, TLR4, RALB
Downregulated			
GO:0,050,853	B cell receptor signaling pathway	0.00953262	CD79B, BLK, CD79A
GO:0,008,277	Regulation of G-protein coupled receptor protein signaling pathway	0.01239782	PDE6H, GRK1, HTR1B, PALM
GO:0,002,429	Immune response-activating cell surface receptor signaling pathway	0.01495808	CD79B, HRAS, NCR3, BLK, CD79A
GO:0,002,757	Immune response-activating signal transduction	0.01811	TNIP3, CD79B, HRAS, NCR3, C1QBP, BLK, CD79A
GO:0,006,974	Cellular response to DNA damage stimulus	0.02079572	RNASEH2A, RAD54L, NHEJ1, PLK1, NEIL1, YAP1, BATF, CDIP
GO:1,901,361	Organic cyclic compound catabolic process	0.02391816	HSD17B14, RNASEH2A, HTR2A, NT5M, ENTPD2, PCBP4
GO:0,044,283	Small molecule biosynthetic process	0.02914066	GAMT, CRBP1, HTR2A, SPHK1, NT5M, ENTPD2, SRR, TNF
GO:0,050,851	Antigen receptor-mediated signaling pathway	0.04083961	CD79B, HRAS, BLK, CD79A
GO:0,044,270	Cellular nitrogen compound catabolic process	0.04267112	RNASEH2A, HTR2A, NT5M, ENTPD2, PCBP4, PDE1B
GO:0,042,493	Response to drug	0.09572823	JUP, RAD54L, HTR1B, INHBA, YAP1, HTR2A, EZH2, TNF

**Table 6:** Upregulated and downregulated biological processes in blood samples of goats transported for 180 min vs. goats not transported (control).

Gene ontology ID	Description	*P*-value	Gene name
Upregulated			
GO:0,006,954	Inflammatory response	1.0804E−06	IL1R2, VNN1, MAPK14, SLC11A1, TLR4, BCL6, C3
GO:0,032,635	Interleukin-6 production	3.5515E−06	IL18RAP, TLR4, C5AR2, MAPK13, IL1RAP, AKIRIN2
GO:0,031,347	Regulation of defense response	5.0556E−06	IL1R2, IL18RAP, MAPK14, TLR4, LTF, BCL6, C3, FGR
GO:0,001,816	Cytokine production	1.7912E−05	IL1R2, CLEC4E, IL18RAP, C5AR1, MAPK14, SLC11A1
GO:0,032,640	Tumor necrosis factor production	7.6694E−05	TLR4, LTF, RARA, C5AR2, CD14, ARG2, TIRAP
GO:0,000,165	MAPK cascade	0.00041843	PTPN1, C5AR1, MAPK14, IGF1R, RAF1, TLR4, CSF1R
GO:0,032,640	Tumor necrosis factor production	0.00007669	TLR4, LTF, RARA, C5AR2, CD14, ARG2, TIRAP, CARD9
GO:0,002,694	Regulation of leukocyte activation	0.00297987	VNN1, LGALS3, TLR4, BCL6, RARA, FGR, CCDC88B
GO:0,031,098	Stress-activated protein kinase signaling cascade	0.00591152	PTPN1, MAPK14, IGF1R, TLR4, LTBR, GADD45A, MAPK13
GO:0,050,778	Positive regulation of immune response	0.00670115	IL18RAP, MAPK14, SLC11A1, TLR4, LTF, RARA, C3
Downregulated			
GO:0,030,903	Notochord development	0.00410461	CRB2, ID3, YAP1
GO:0,030,199	Collagen fibril organization	0.00233333	COL1A2, SERPINF2, COL1A1, DDR2
GO:2,000,146	Negative regulation of cell motility	0.00589863	GPR18, JUP, PHLDB2, MARVELD3, SPINT2, DUSP10
GO:006,140	Regulation of nucleotide metabolic process	0.00657536	ATP5IF1, PDE4D, IP-10, ME1, PARP1
GO:0,032,496	Response to lipopolysaccharide	0.00687099	CD96, CD6, DUSP10, PDE4D, TNFRSF11A, CCL2
GO:1,902,547	Regulation of cellular response to vascular endothelial growth factor stimulus	0.00196244	DCN, ADAMTS3, JCAD
GO:0,045,879	Negative regulation of smoothened signaling pathway	0.0020109	CD3E, IFT122, TULP3, MOSMO
GO:0,007,254	JNK cascade	0.00223223	MARVELD3, DUSP10, ULK4, ERN2, COPS5, SERPINF2
GO:0,051,090	Regulation of DNA binding transcription factor activity	0.00335442	OTULIN, JUP, ID3, PRKCQ, GTF2A2, COPS5, ADORA3
GO:0,001,568	Blood vessel development	0.00368768	OTULIN, JUP, BAK1, NSDHL, COL1A2, PDCL3, TBX2

### KEGG

**Figure 7:**
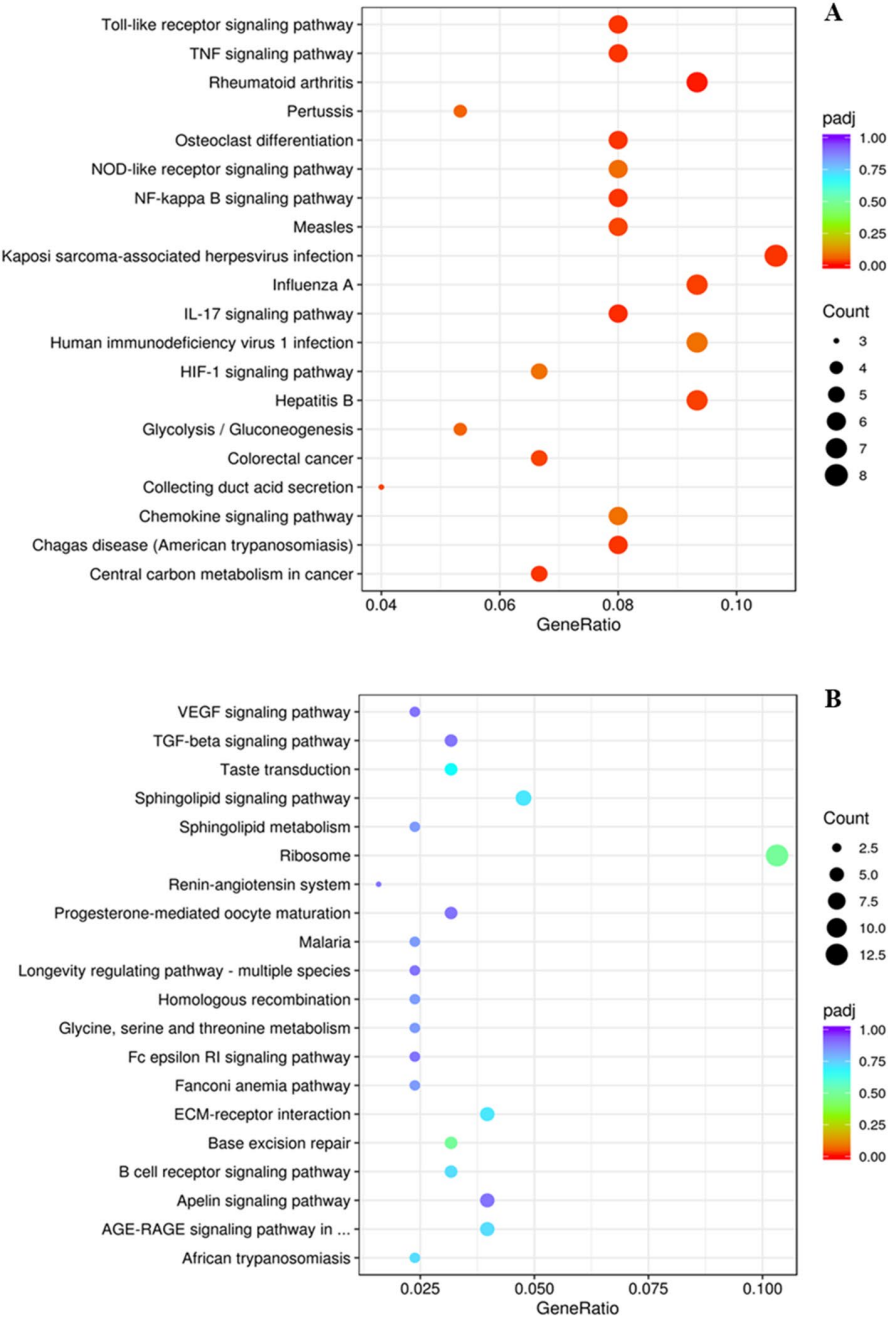
(**A**) Upregulated and (**B**) downregulated KEGG pathways^27^ in blood samples from goats subjected to control (not transported but held in pens) versus 30 min (transported for 30 min) stress treatments.

**Figure 8:**
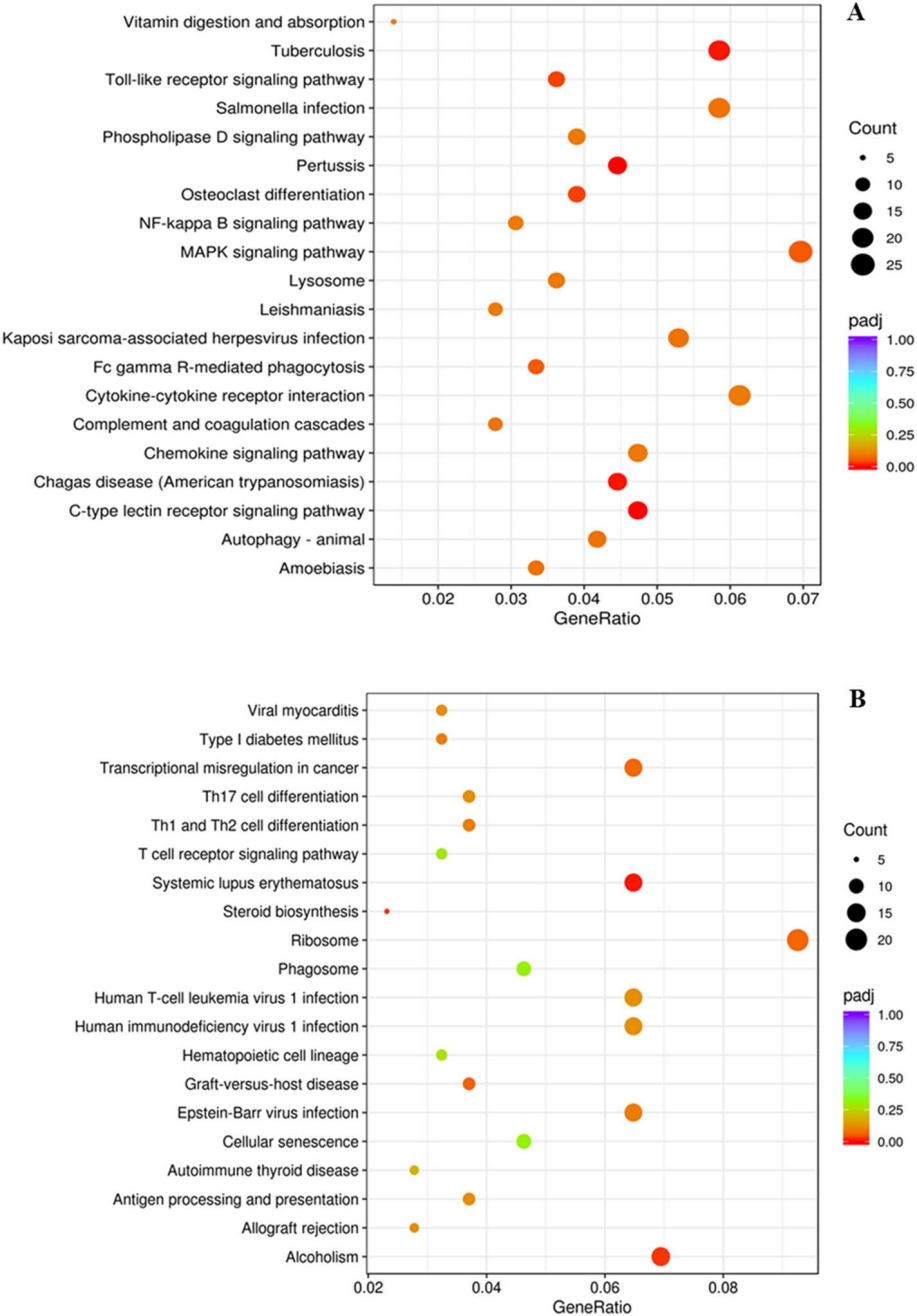
(**A**) Upregulated and (**B**) downregulated KEGG pathways^27^ in blood samples from goats subjected to control (not transported but held in pens) versus 180 min (transported for 180 min) stress treatments.

**Figure 9:**
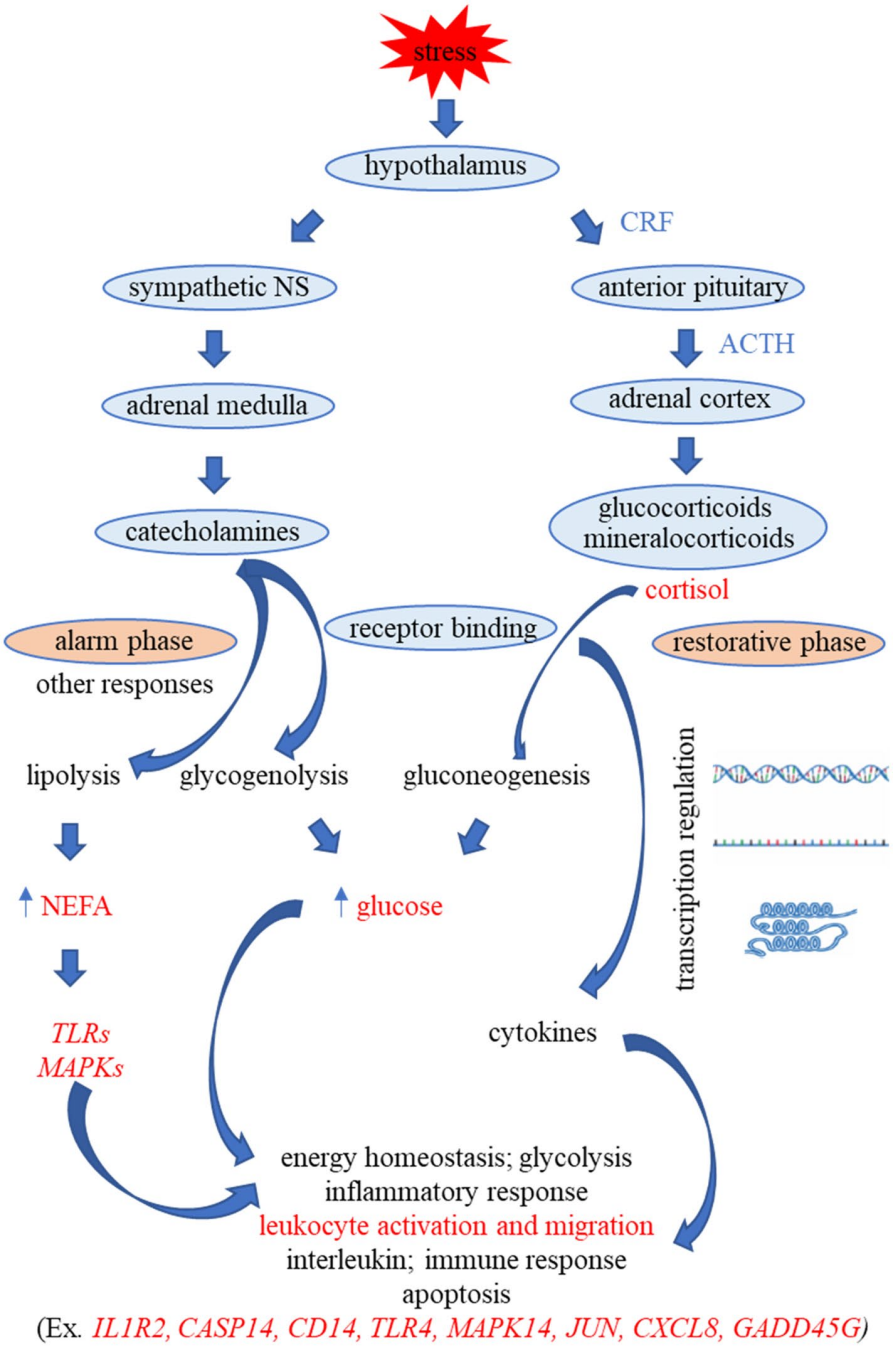
A mechanism model showing the possible relationships among the physiological stress responses measured and differentially expressed genes identified in the blood of goats in this study.

The pathway analysis of DEGs related to transportation stress was done using KEGG database. All DEGs obtained from DESeq2 method were mapped to KEGG pathways to determine genes and pathways related to stress. For 30 min versus control comparison, IL-17 signaling pathway, NF-kappa B signaling pathway, chemokine signaling pathway, glycolysis/gluconeogenesis pathways, and toll-like receptor signaling pathway were among those upregulated and significantly enriched (Fig. 7A). Some of the downregulated pathways significantly enriched for this comparison were B cell receptor signaling pathway, glycine, serine, and threonine metabolism, VEGF signaling pathway (vascular endothelial growth factor), TGF beta signaling pathway (transforming growth factor beta), and AGE (advanced glycation end products)—RAGE (receptor for advanced glycation end products) signaling pathway (Fig. 7B). Comparison of 180 min blood versus control group showed that pathways such as toll-like receptor pathway, phospholipase D signaling pathway, NF-kappa B signaling pathway, MAPK signaling pathway, and chemokine signaling pathway were upregulated (Fig. 8A), and steroid biosynthesis, antigen processing and presentation, T cell receptor signaling pathway, phagosome, and Th1 and Th2 cell differentiation were downregulated (Fig. 8B). The possible relationships among the phenotypic variables determined and the DEGs and pathways identified in response to acute stress in this study are depicted in Fig. 9.

## Discussion

Stress stimulates the hypothalamus–pituitary–adrenal (HPA) axis and sympatho-adrenal axis which activates the adrenal gland to release stress hormones such as epinephrine, norepinephrine, and cortisol^28,29^. An increase in plasma cortisol concentration is a good indicator of transportation stress in goats^3,8,9,30^. Studies in other livestock species have also shown transportation stress causes an increase in cortisol levels^31–33^. In the current study, plasma cortisol concentrations were higher in 30 min and 180 min groups compared to the control group of goats. These results are consistent with the earlier reports that cortisol level increases with increasing transportation time^3,9,34^. However, Bulitta et al.^35^ reported that when cows and bulls were transported for up to 12 h, cortisol concentrations were the highest at a shorter transportation time of 4 h and lowest at 12 h of transportation due to habituation.

Stress hormones act on the receptors through a series of genes and pathways that influence energy homeostasis. Glucocorticoids play an important role in the regulation of carbohydrate, protein, and fat metabolism^36^. Cortisol stimulates gluconeogenesis in the liver and helps in the maintenance of blood glucose levels by breaking down amino acids, glycerol, lactate and propionate^37^. The complexes that are formed when corticosteroids bind to the glucocorticoid receptors in the cytoplasm are transported to the nucleus to act as transcription factors to modulate gene expression^38,39^.

Plasma glucose levels are used as indicators of stress in small ruminants^40^. Stress stimulates the secretion of epinephrine from the adrenal medulla, which activates glycogenolysis in the liver and muscle. Transportation stress has been reported to elevate plasma glucose levels, primarily due to the breakdown of glycogen in the liver^41^. In our study, glucose concentrations increased with increasing transportation time. The glucose response seen in this study is consistent with those in earlier studies conducted on transportation effects in cattle and bulls^35,42^.

In addition to increasing plasma glucose concentrations during stress, catecholamines also stimulate lipolysis resulting in increases in plasma NEFA concentrations^9,43^. Plasma NEFA concentrations were reported to be higher in Holstein heifers transported for 100 km and 200 km than in a control group of heifers^43^. In our study, NEFA levels in both 30 min and 180 min groups were higher than the control group. Other stressors also cause increases in NEFA concentrations in goats, as feed deprivation has been reported to increase plasma NEFA concentrations^44^. An increase in NEFA concentration has been suggested to activate signaling pathways that regulate gene expression, in addition to their role in providing energy^45^. Plasma CK activity increases in response to stress, muscular activity, and/or muscle damage in animals^3^, although in the present study, CK activities were not significantly affected by transportation. Transportation itself is unlikely to cause increases in plasma CK activities; however, vigorous physical activities such as herding, loading, and unloading are more important in determining plasma CK levels in goats. Agonistic encounters, particularly when goats are horned, are likely to cause muscle damage and elevation of CK activities. The goats used in the present experiment were horned Spanish goats; however, behaviors were not observed during transportation. Kannan et al.^9^ also did not see an increase in plasma CK levels after transportation in Alpine goats.

One of the most significant effects of stress in animals is that it suppresses and weakens the immune system^29^. Stress affects the immune system through the actions of glucocorticoids and catecholamines. Glucocorticoids are known to act on the T helper cells resulting in type 1/type 2 cytokine production, which alters the balance of cellular and humoral immunity and reduces immune function in the body^46^. In the present study, the N/L ratio was higher in the 180 min group compared to 30 min or control groups due to an increase in neutrophil counts and decrease in lymphocyte counts. A similar effect has been previously reported in Spanish goats^3^. Zulkifli et al.^34^ noted that goats transported by road under hot and humid tropical conditions showed drastically altered N/L ratios. A large number of DEGs related to inflammatory response such as *IL1R2, MAPK14, TLR4, CCL24, CXCL8,* and *BCL6* were identified in our treatment comparisons. Leukocyte activation and proliferation (ex. *JUN, CXCL8, TLR4*) was among the significantly upregulated biological process identified in our study. The *IL1R2* gene was found to be significantly upregulated in goats in the 180 min versus control comparison, which is also involved in biological processes such as regulation of defense mechanism and cytokine production, in addition to inflammatory response. There are two subtypes of interleukin type 1 receptors, *IL1R1* and *IL1R2*, and both have significant effects on immune response and inflammatory reactions^47^. Interleukin 8 (*CXCL8*) and *CCL24* are chemokines known to aid chemotaxis in leukocytes. Chemokines could cause the recruitment of neutrophils and lymphocytes to affected tissues since stress transcriptionally triggers inflammatory reactions^48^. Fernando et al.^49^ reported that *BCL6* (B-cell lymphoma 6) has evolved to enable stress tolerance in vertebrates as a part of the heat shock factor 1 (*HSF1*)-induced response to stress. Zhao et al.^50^ suggested that *JUN* and *CXCL10* could be among the molecular biomarkers for transportation stress in cattle. The *JUN* gene is involved in multiple pathways, including B cell function, proliferation, and survival as well as apoptosis^51^. Based on the DEGs identified in response to transportation stress in goats in our study along with those reported in cattle^50^, *JUN* and *CXCL* gene family could also be biomarkers for transportation stress in sheep. While animals can withstand mild to moderate stress due to stimulation of the immune system, severe stress is likely to have a negative effect on the animal’s ability to fight infections^14^.

The DEGs enriched related to stress response were inflammatory response, apoptotic response, exosome (RNase complex), kinase activity, and cytokine receptor binding in our study. Similar DEGs were identified in pigs subjected to cortisol treatment^12^. The release of short- and long-acting stress hormones and their actions on receptors in cells are important factors that initiate the change in gene expression and alter cellular homeostasis in response to stress^52^. Specifically, cortisol and catecholamines modulate immunity by acting on the receptors on antigen presenting cells that either releases or inhibits the mediators of inflammation and immunity^53^. Glucocorticoids are also involved in the apoptosis mechanism^54^ through the activation of caspases^55^. The caspase family of genes are involved in signaling pathways of apoptosis, necrosis, and inflammation^56^, and the DEGs related to inflammation are also invariably involved in cell apoptosis. The nuclear factor kappa B (*NF- k B*) is a transcriptional factor, which is a primary regulator of stress response that is activated by *TLRs* through MyD88-dependent signaling pathway^57^, inducing apoptosis^58^. Our study also revealed that *CASP14* expression was significantly upregulated among the DEGs, in addition to genes such as *TLR4, TLR7, NFKBIA* (nuclear factor of kappa light polypeptide gene enhancer in B-cells inhibitor alpha), *CEBPB* (CCAAT/enhancer-binding protein beta), *JUN, GADD45G, DDIT4* (DNA damage-inducible transcript 4)*, MAPK4* that are related to both inflammation and apoptosis. Growth arrest DNA damage 45 (*GADD45G*) genes are involved in stress signaling in response to physiological or environmental stressors resulting in cell cycle regulation, DNA repair, and apoptosis^59^. There is further evidence in our study that stress activates TLRs and related signaling pathways in goats, as increased NEFA concentrations have been suggested to stimulate *TLRs*^60^.

Gene ontology terms also showed that several biological processes related to energy metabolism were upregulated (ex. response to oxidative stress, stress-activated protein kinase signaling cascade, stress-activated *MAPK* cascade). Enriched KEGG pathways were mainly related to stress response, inflammatory response, apoptosis pathways, glycolysis and gluconeogenesis, and chemokine signaling pathway. Previous studies on transcriptome profiling in goats and cattle have shown that wingless-type MMTV integration site family (Wnt), MAPK, and PI3K-Akt signaling pathways play an important role in stress and apoptosis^61^. Both the activation and the effects of MAPK signaling appear to be complex phenomena. While the MAPK signaling pathway regulates stress responses and influences lipid metabolism^62^, stress-activated MAPK signal transduction has also been reported to prevent cell growth and promote apoptosis^63^. Akira et al.^64^ reported that the *TLRs* stimulate complex intracellular signaling resulting in the activation of the *MAPKs* and transcription factors. Furthermore, Zbinden-Foncea et al.^61^ found evidence in mice that circulating NEFA concentrations stimulate *MAPK* through the activation of *TLRs* in times of energy demand, although the authors emphasized the need for more elevation of NEFA concentrations played a role in the activation of both *TLRs* and *MAPKs*. Studies conducted in magpie birds and duroc pigs have further confirmed that MAPK pathway plays an important role in regulating energy homeostasis and physiological stress responses^65,66^.

Transcriptomic profiling in concert with phenotypic measures and other approaches such as metabolomic analysis can complement the data and allow for a better comprehension of the effects of stress in livestock. This study provided valuable information on the possible molecular mechanisms involved in response to transportation stress in Spanish goats. As shown in Fig. 9, the activation of adrenal cortex in response to transportation stress results in the release of glucocorticoids that bind to specific receptors in the cytoplasm. These complexes are then transported from cytoplasm to the nucleus to act as transcription factors to modulate gene expression (ex. *JUN, CXCL8, IL1R2, GADD45G, NFKBIA, CASP14*) via activation of cytokines, and thus regulate leukocyte, immune, inflammatory, and apoptotic responses. The effect on leukocytes is reflected phenotypically by an increase in N/L ratio as observed in the present experiment. The increase in plasma NEFA concentrations activates both TLRs and MAPKs that regulate energy homeostasis pathways during stress among other functions. With the further characterization of DEGs in response to transportation, it is possible to identify novel biomarkers for stress in goats that will enable development of better husbandry practices.

## Conclusions

We analyzed the effect of transportation on stress phenotypically and using RNA-seq methodology to further understand the intrinsic mechanism of changes taking place at the molecular level. Specifically, we focused on the genes and pathways related to stress, inflammation, energy homeostasis, and cell apoptosis. Stress levels were higher in the transported group compared to the non-transported controls as confirmed by higher concentrations of cortisol, glucose, and NEFA in the transported goats. In addition, lymphocyte counts decreased, and neutrophil counts and N/L ratios increased due to stress in goats, possibly reducing the body’s ability to fend off infections. The DEGs related to inflammatory pathways, caspases, and apoptosis were highly expressed in the transported group of goats compared to non-transported goats. Several DEGs related to inflammatory response such as *IL1R2, CASP14, CD14, TLR4, and MAPK14* were identified in our comparisons. Stress hormones could induce apoptosis by activating TLRs, caspases, and related signaling pathways. Stress-related increases in plasma NEFA concentrations likely activate MAPK signaling through stimulation of TLRs. Stress in goats leads to a sequence of events at cellular and molecular levels that are related to the changes in blood metabolites, energy metabolism, leukocyte counts, and apoptosis. Although further confirmations are warranted, the RNA-seq analysis in this study enabled us to determine the levels of mRNA in the blood of stressed goats and identify DEGs leading to a better understanding of the biological pathways affected. The results of this study may be useful in developing suitable targeted management methods, from antioxidant feed supplement to selection of animals for breeding, that will help minimize the negative impacts of transportation stress on health, welfare, and productivity.

## Supplementary Information

Supplementary Information.

## Data Availability

The raw data and processed data have been submitted to the Gene Expression Omnibus (GEO). The accession number is GSE210841.
