# Supplementary figures and images for: Differential gene expression analysis using RNA-seq in the blood of goats exposed to transportation stress
Source: Sci Rep. 2023 Feb 3;13:1984. doi: 10.1038/s41598-023-29224-5 (PMC9898539; doi:10.1038/s41598-023-29224-5)

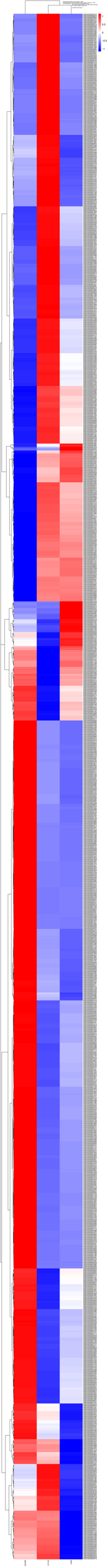

Supplement: Supplementary file 1 — Supplementary Information. [file 41598_2023_29224_MOESM1_ESM.pdf]
